# Helical Electron Beam Micro‐Bunching by High‐Order Modes in a Micro‐Plasma Waveguide

**DOI:** 10.1002/advs.75489

**Published:** 2026-04-30

**Authors:** Xingju Guo, Longqing Yi

**Affiliations:** ^1^ State Key Laboratory of Dark Matter Physics, Key Laboratory for Laser Plasma (Ministry of Education), Tsung‐Dao Lee Institute & School of Physics and Astronomy Shanghai Jiao Tong University Shanghai China; ^2^ Collaborative Innovation Center of IFSA (CICIFSA) Shanghai Jiao Tong University Shanghai China

**Keywords:** helical electron beam, high‐order waveguide mode, relativistic laser‐plasma interaction

## Abstract

Electron acceleration by a high‐power Laguerre‐Gaussian pulse in a micro‐plasma waveguide is investigated. When the incident laser travels in the waveguide, electrons on the wall are extracted into the vacuum core and accelerated by the longitudinal field of the waveguide mode. Using 3D particle‐in‐cell simulations, we demonstrate that circularly polarized Laguerre–Gaussian laser pulses can excite high‐order waveguide modes that exhibit helical longitudinal electric fields. The 3D profile of this accelerating field is imprinted into the high energy electron beam, leading to customized helical micro‐bunching of high‐energy (∼ GeV) electron beams with extremely high charge (100s nC), and small divergence ($∼2^{\circ }$). In particular, we demonstrate that when co‐propagating with high‐order waveguide modes, the electron can migrate transversely to keep up with accelerating brackets, leading to significant boost of the acceleration energy. This work paves the way towards the generation of high‐charge, relativistic electron beams with controlled helicity, which holds great potential for advances in fundamental science and a variety of applications.

## Abstract 2

When a circularly polarized high‐power Laguerre–Gaussian laser beam travels in a micro‐plasma waveguide, it excites azimuthal modes exhibiting helical longitudinal electric fields. The 3D profile of this accelerating field is imprinted into the high energy electron beam, leading to customized helical micro‐bunching of high‐energy (∼ GeV) electron beams with extremely high charge (10s nC), and small divergence ($∼2^{\circ }$).

## 1 Introduction

Laser plasma accelerators can sustain large acceleration gradients up to hundreds of ${\mathrm{GVm}}^{-1}$, orders of magnitude greater than that of a state‐of‐the‐art radio‐frequency accelerator [1, 2]. This makes them an attractive option for developing compact, table‐top accelerators, which is key to improving accessibility to high‐energy electron beams for a variety of applications in industry, medicine and fundamental research. Among these laser‐based accelerating schemes, laser wakefield acceleration (LWFA) [3] can produce multi‐GeV, quasi‐monoenergetic electron beams with high quality [4, 5, 6, 7], making it possible for driving X‐ray free electron lasers [8, 9, 10, 11]. On the other hand, self‐modulated LWFA in near‐critical‐density plasma [12, 13, 14] and direct laser acceleration (DLA) [15, 16, 17, 18, 19, 20, 21, 22, 23, 24, 25, 26] are often relied on to generate electron beams with high charge up to hundreds of μC, broad‐band energy spectra and relatively‐large divergence, such electron beams are of interest for developing positron sources [27, 28, 29], as well as producing high‐power Terahertz pulses via coherent transition radiation [15, 30].

High‐power laser pulse interacting with a micro‐plasma waveguide (MPW), provides a promising alternative for tabletop electron accelerator as the beam parameters fill in the gap between the aforementioned schemes. The MPW is made of solid cladding (plastic or metal) and hollow core, with a diameter comparable to laser wavelength (∼10μm). Previous works [31, 32, 33, 34] have shown it can guide the incident high‐power laser pulse as *waveguide modes* over long distances beyond the Rayleigh range. The maximal acceleration energy is typically a few hundreds of MeVs, with broad‐band energy spectra and good directionality. A total charge of a few tens of nCs can be obtained, as these electron bunches are extracted from solid waveguide walls, such that they inherit the high‐density nature [35]. These features have been demonstrated experimentally [36] and utilized to produce ultra‐intense Terahertz pulses [37].

In general, electron acceleration via laser‐MPW interaction also falls into the DLA category. But unlike so‐called “vacuum laser acceleration,” where the energy gain is mostly attributed to the work done by transverse electric field, it has been established that in an MPW the electrons are accelerated predominately by the longitudinal electric field of waveguide modes [32, 38]. The underlying physical processes, such as dephasing effect due to superluminal phase velocity of the modes, remain largely unexplored even theoretically. In particular, so far the theories only consider the fundamental waveguide modes which have the smallest eigenvalue, electron acceleration by high‐order modes have never been studied from first principles.

Moreover, since the electrons can only be accelerated in suitable phases of the waveguide modes [31], the electron beam is thus shaped by the electromagnetic structure of the waveguide modes [35, 39, 40, 41], which can be leveraged to enable controlled helical micro‐bunching. To achieve such capability is very challenging for LWFA, as a “light spring” is required to transfer orbital angular momentum (OAM) to the plasma wave [42]. On the other hand, DLA studies with twisted laser field [22, 24, 25] mostly focus on a zero‐OAM mode where acceleration is maximal but helical micro‐bunching is not present.

To achieve full degree‐of‐freedom manipulation of the micro‐bunching process, the capability of exciting arbitrary high‐order waveguide modes is necessary. In this work, we show that by employing a circularly polarized (CP) Laguerre–Gaussian (LG) pulse as the driver, one can selectively generate high‐order azimuthal modes in the MPW, which can be used to induce controlled helical micro‐bunching of electron beams, by varying the topological charge of the LG pulse. We further show that the dephasing effect, which is expected to be very severe for the high‐order modes (because their phase velocities are substantially above light speed), can be mitigated drastically by the azimuthal migration of the accelerated electrons, such that the cutoff energy is almost invariant for helical bunches with different helicity. This approach grants the capability of producing ultra‐dense relativistic electron beams with arbitrarily customized helical micro‐bunching, and a maximum energy of a few GeV, total charge above 200 nC, divergence of $∼2^{\circ }$, which is valuable for fundamental science [43] and a variety of applications [44, 45, 46, 47].

## 2 Theoretical Model for High Order Waveguide Modes

We first present our theory on high‐order waveguide modes. When the drive pulse is relativistically intense, a strong evanescent wave is present at the plasma‐vacuum interface. The co‐moving evanescent wave modifies the boundary condition [48] and effectively determines the waveguide modes that can propagate in a MPW. This is fundamentally different from conventional metallic waveguides, By considering the plasma response to the propagating electromagnetic wave, the eigenvalue equation can be obtained as [49]

(1) $$A\left(m\right)\left[B\left(m\right)-2C\left(m\right)\right]+\frac{\omega_{p}^{2}}{\omega_{0}^{2}}C\left(m\right)K\left(m\right)=0,$$

where

(2) $$\begin{array}{c} A(m)=J(m)+K(m), \end{array}$$

(3) $$\begin{array}{c} B\left(m\right)=A\left(m\right)+\frac{\omega_{p}^{2}}{\omega_{0}^{2}}\left[\frac{m}{y^{2}}-K\left(m\right)\right], \end{array}$$

(4) $$\begin{array}{c} C\left(m\right)=\frac{m}{x^{2}}+\frac{m}{y^{2}}, \end{array}$$

(5) $$\begin{array}{c} J\left(m\right)=\frac{J_{m+1}\left(x\right)}{xJ_{m}\left(x\right)},\;K\left(m\right)=\frac{K_{m+1}\left(y\right)}{yK_{m}\left(y\right)}. \end{array}$$

Here $\omega_{0}$ is the laser frequency, $\omega_{p}=\sqrt{4\pi n_{p}e^{2}/m_{e}}$ is the plasma frequency of the cladding, and $y=\sqrt{{\left(cr_{0}/\omega_{p}\right)}^{2}-x^{2}}$, where c, e, $m_{e}$, $n_{p}$ and $r_{0}$ denote vacuum light speed, elementary charge, electron mass, plasma density and MPW radius, respectively. Specifically, A(m), B(m), C(m) are just dimensionless functions that are defined for simplicity, $J_{m}\left(x\right)$ is the Bessel function of the first kind, and $K_{m}\left(y\right)$ is the modified Bessel function of the second kind.

The eigenvalue equation Equations ((1), (2), (3), (4), (5)) was first derived in Ref.  [49] to study the transfer of electromagnetic waves, and has previously been applied in the study of electron acceleration [40] and X‐ray emission [31, 32], but the implications for high‐order waveguide modes and their application have never been discussed. By solving Equations ((1), (2), (3), (4), (5)), one obtains the roots $x_{|m|,n}$, where the subscript |m| denotes the absolute value of the azimuthal mode number, and n is the radial mode number, namely, $x_{|m|,n}$ is the nth root for a given |m|. The transverse wavenumber is thus obtained as $k_{T}=x_{|m|,n}/r_{0}$, which satisfies $k_{0}=\omega_{0}/c=\sqrt{k_{x}^{2}+k_{T}^{2}}$.

**FIGURE 1:**
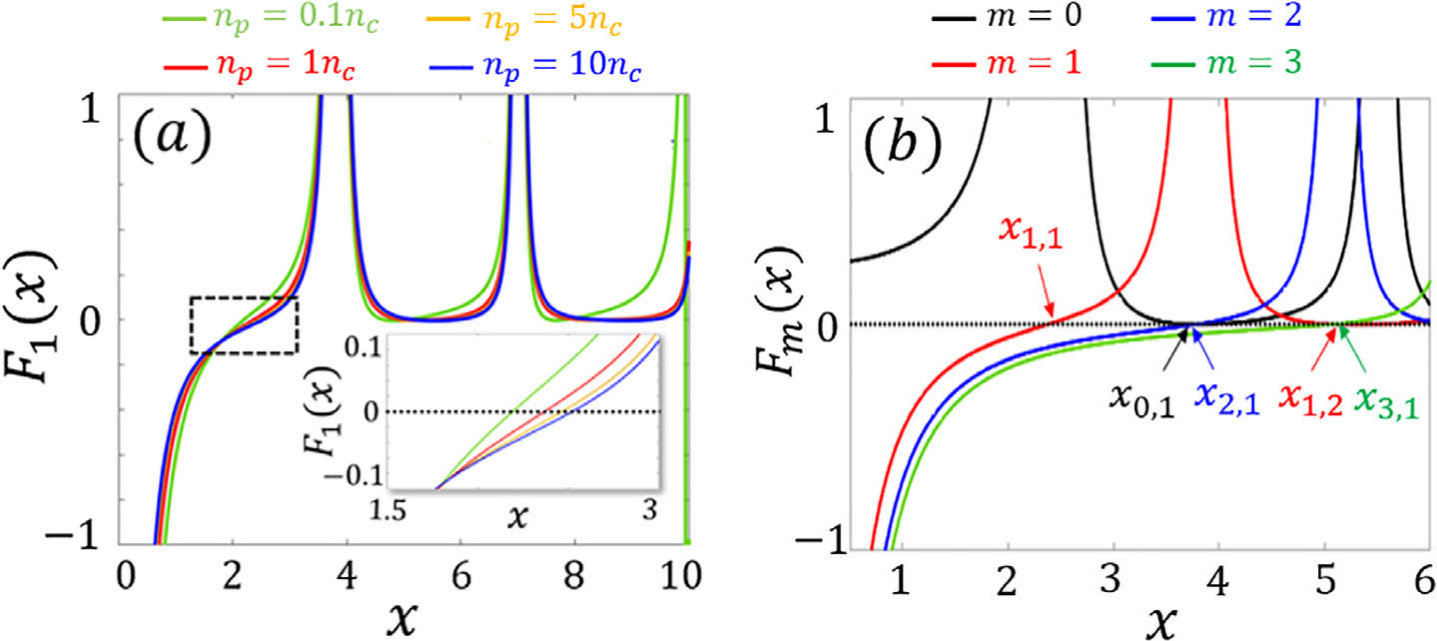
(a) Left‐hand side of eigenvalue equation $F_{m}\left(x\right)\equiv A\left(m\right)\left[B\left(m\right)-2C\left(m\right)\right]+\left(\omega_{p}^{2}/\omega_{0}^{2}\right)C\left(m\right)K\left(m\right)$ is plotted against x for different plasma density $n_{p}$ with when m=1. The inset shows a zoom‐in of the area inside the black dashed lines. (b) $F_{m}\left(x\right)$ plotted for different m, the plasma density is fixed at $n_{p}=1n_{c}$. The black dotted lines in (a, b) indicates the roots of eigenvalue equations $F_{m}\left(x\right)=0$, and eigenvalues $x_{1,1},x_{0,1},x_{2,1},x_{1,2}$ and $x_{3,1}$ are shown in (b).

Before proceeding to the high‐order waveguide mode and helical electron acceleration, we first discuss two important features of eigenvalue equation Equation ((1), (2), (3), (4), (5)):

(i) The value of $x_{|m|,n}$ does not depend sensitively on the plasma density $n_{p}$ in the cladding, as long as $n_{p}$ is overdense. This is shown in Figure 1a, where the left‐hand side of Equation (1) is plotted against x for various plasma densities $n_{p}$, the black dashed line in the inset indicates the eigenvalues $x_{1,n}$ for each case. One can see that when $n_{p}>n_{c}$, the value of $x_{1,n}$ varies little for different values of $n_{p}$. Note that we restrict the discussion to $n_{p}\leq 10n_{c}$, because inside the micro‐plasma waveguide (MPW), the laser light is grazing incident on the plasma wall, and is therefore unlikely to penetrate into high‐density regions beyond $10n_{c}$.
(ii) The left‐hand side of Equation (1) is an even function of m. Therefore, normally one does not distinguish the sign of m, however, in this study we find the sign of m is important because it is related to the handedness of the helical micro‐bunching, and to the excitation of high‐order radial modes n>1 (discussed later). In Figure 1b, we show the lowest value of eigenvalues with m=0,±1,±2,±3, which will be discussed in the remainder of this work. One can see that they satisfy: $x_{1,1}<x_{2,1}\approx x_{0,1}<x_{1,2}\approx x_{3,1}$.

Since both electric and magnetic fields are present in the propagation direction, the waveguide mode can be denoted as ${\mathrm{HE}}_{|m|,n}$. We write the electric field of the waveguide mode in Cartesian coordinates with +x as the propagating direction.

(6) $$\begin{array}{cc} E_{x}= & \frac{k_{T}}{k_{0}}E_{m}J_{m}\left(k_{T}r\right)\\ & \times \exp(ik_{x}x-i\omega_{0}t+i\sigma m\phi -i\frac{\pi }{2}), \end{array}$$

(7) $$\begin{array}{cc} E_{⊥}\approx & \left(e_{y}+i\sigma e_{z}\right)E_{m}J_{m-1}\left(k_{T}r\right)\\ & \times \exp\left[ik_{x}x-i\omega_{0}t+i\sigma \left(m-1\right)\phi \right], \end{array}$$

where $E_{m}$ is the amplitude of the transverse electric field of the mode, $r=\sqrt{y^{2}+z^{2}}$ is the radial coordinate, and ϕ is the azimuthal angle with respect to the y axis. The polarization of the drive laser is controlled by σ=+1 and −1, corresponding to right‐handed CP (RCP) and left‐handed CP (LCP) polarization, respectively. Note that both $E_{x}$ and $E_{⊥}$ exhibit helical phase fronts, which depend on the spin σ and azimuthal mode number m.

Because the generated waveguide modes must the satisfy initial condition, the field structure at the entrance of the MPW is determined by the incident laser field $E_{l}$. In particular, the terms depending on ϕ in Equation (7) must be the same as those in $E_{l}$. This explains why previous studies [31, 32] employing a planar wave as the driver only consider modes with m=1.

To excite high‐order azimuthal modes (m≠1), we consider a circularly polarized LG pulse as the driver [50]:

(8) $$\begin{array}{cc} E_{l} & =\left(e_{y}+i\sigma e_{z}\right)E_{0}{\left(\frac{\sqrt{2}r}{w_{0}}\right)}^{l}\exp\left(\frac{-r^{2}}{w_{0}^{2}}\right)\sin^{2}\left(\frac{\pi t}{2\tau_{0}}\right)\\ & \;\times \exp(ik_{0}x-i\omega_{0}t+il\phi ), \end{array}$$

where $E_{0}$, $w_{0}$, $\tau_{0}$, and l represent the amplitude, focal spot size, duration, and topological charge of the driver, respectively. By equating the ϕ‐dependent terms in Equations (7) and (8), one can readily obtain the azimuthal mode number that satisfies the initial condition

(9) $$\begin{array}{c} m=(l+\sigma )\sigma . \end{array}$$

Here, both the topological charge (l) and polarization (σ) play a role, because the helical electron beam must be produced by an accelerating field (Equation 6) with continuous rotational symmetry. Therefore, the LG drive laser must be circularly polarized to satisfy this condition. In addition, it can be shown that a linearly polarized LG pulse leads to double modes excitation with different azimuthal numbers, because it can be decomposed into RCP and LCP components (see Section 4.2).

Finally, we note that due to the mismatch between the drive laser pulse and the waveguide mode at the entrance, typically multiple modes (${\mathrm{HE}}_{|m|,n}$) with the same azimuthal number (m) but different radial number (n) are excited. The interference between these modes results in periodic focusing and defocusing oscillations of the electromagnetic wave in the channel. Since the helical micro‐bunching is controlled solely by m, and the maximum electron acceleration energy is determined by the lowest radial mode with the smallest phase velocity, the excitation of high‐order radial modes does not affect our key results. We therefore consider only the radial mode with the lowest n for simplicity. A brief discussion is given in the Supplemental Materials.

## 3 Simulations Results

**FIGURE 2:**
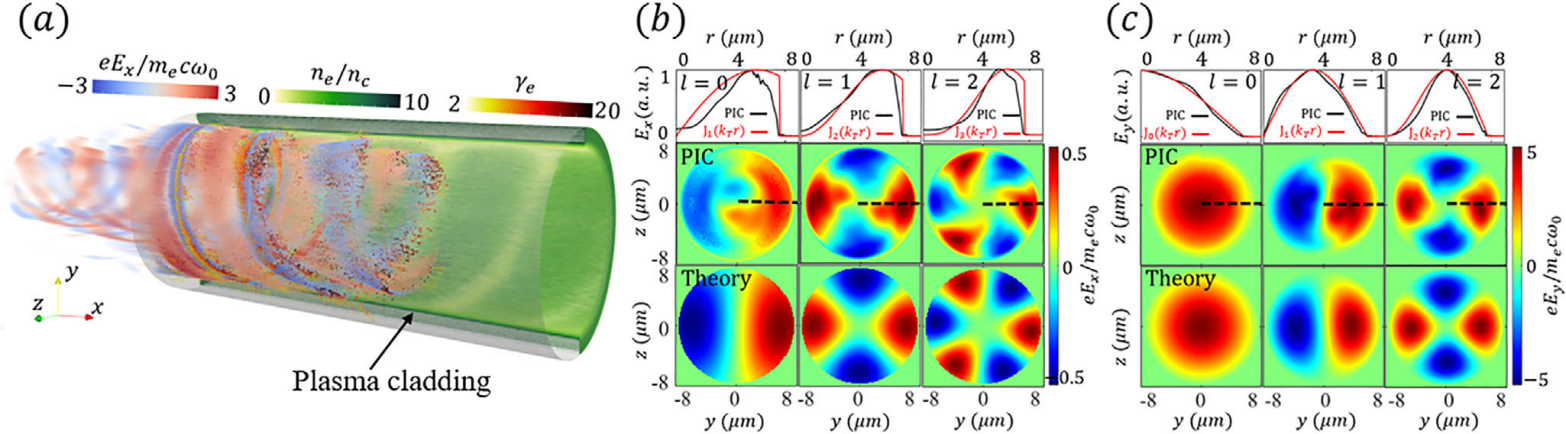
(a) Schematic sketch of the waveguide mode excitation. The 3D color‐coded acceleration field $E_{x}$ (blue‐red, in unit of $m_{e}c\omega_{0}/e$); and surface electron density fluctuation (white‐green, normalized to the critical density $n_{c}$). The orange dots represents the injected electrons, and the color shows the Lorentz factor of each individual electron. The drive laser pulse is RCP with l=1. The distribution of (b) $E_{x}$ and (c) $E_{y}$ fields, where the first, second, and third columns corresponding to topological charges l=0,1,2 of the incident laser pulse. The second and third row show the results from PIC simulations and Equations (6) and (7), respectively. In the first row of (b) and (c), the black lines illustrate the radial distribution of the fields in each case along the dashed lines, and the red lines show the prediction of the theory.

In the following, we demonstrate the generation of high‐order waveguide mode with 3D PIC simulations. Figure 2a illustrates the longitudinal electric field produced at the entrance when a RCP (σ=1) LG beam with topological charges l=1 enters the MPW (radius $r_{0}=8\;\mu m$). The normalized laser amplitude adopted in the simulations is $a_{0}=eE_{0}/m_{e}\omega_{0}c=15$, corresponding to an intensity of $I_{0}=6.2\times 10^{20}$ W/${\mathrm{cm}}^{2}$, the laser wavelength is $\lambda_{0}=1\;\mu m$, and spot size $w_{0}=0.8r_{0}=6.4\;\mu m$, pulse duration $\tau_{0}=42$ fs (FWHM is 30 fs). The laser enters a pre‐ionized MPW [assumed plastic (CH)] at $x_{0}=5\;\mu m$, and propagates in the +x direction. The electron density in the MPW cladding is $n_{0}=20n_{c}$, where $n_{c}=m_{e}\omega_{0}^{2}/4\pi e^{2}\approx 1.1\times 10^{21}$ ${\mathrm{cm}}^{-3}$ is the critical density. A density gradient is present at the inner surface to account for the plasma expansion due to heating by the prepulse, $n_{p}\left(r\right)=n_{0}\exp\left[\left(r-r_{0}\right)/h\right]$ for $r_{0}-2\;\mu m<r<r_{0}$, with h=0.2μm being the scale length. The simulation is performed with the fully kinetic PIC code epoch [51]. The size of the simulation box is $L_{x}\times L_{y}\times L_{z}=32\times 18\times 18\;{\mu m}^{3}$, which is sampled by 1280×360×360 cells, with 4, 2, and 2 macro‐particles per cell for electrons, $C^{6+}$ and protons, respectively. Specifically, open boundary condition is applied in the PIC simulations, and a moving window is employed to improve computational efficiency. Numerical convergence for 3D PIC simulations has been tested with results shown in Supplemental Materials.

As a primary example, Figure 2a shows the color‐coded longitudinal electric field and the electron density fluctuation (evanescent wave) near the entrance of the MPW. A significant increase of the $E_{x}$ field strength can be observed as the laser enters the MPW, which is important for electron acceleration. Moreover, the laser longitudinal field exhibits double‐helix structure, as predicted by Equation (9). The density fluctuation shown by the white‐green, matches the $E_{x}$ field structure on the wall, indicating that the boundary condition is satisfied.

For the transverse distribution of $E_{x}$ and $E_{y}$ fields generated by three LG lasers with different topological charges l=0,1,2 (all are RCP with σ=1), we obtain m=1,2,3, as shown in Figure 2b,c, which agrees very well with the theory. Notably, unlike in the conventional metallic waveguide, the $E_{x}$ field is nonzero at the MPW boundary $r=r_{0}$ (first row of Figure 2b). Instead, it drops abruptly to zero within a short distance, indicating a strong evanescent wave is present.

We next examine the radial profile of the electromagnetic fields in the MPW. Equations (6) and (7) suggest the longitudinal and transverse electric fields of azimuthal mode number m are proportional to $J_{m}\left(k_{T}r\right)$ and $J_{m-1}\left(k_{T}r\right)$, respectively. This relationship is tested in the first row of Figure 2b,c. Moreover, the second and third rows present a comparison of the transverse electric field profiles between the PIC simulation data and those calculated from Equations (6) and (7). Evidently, the theory matches the data very well.

**FIGURE 3:**
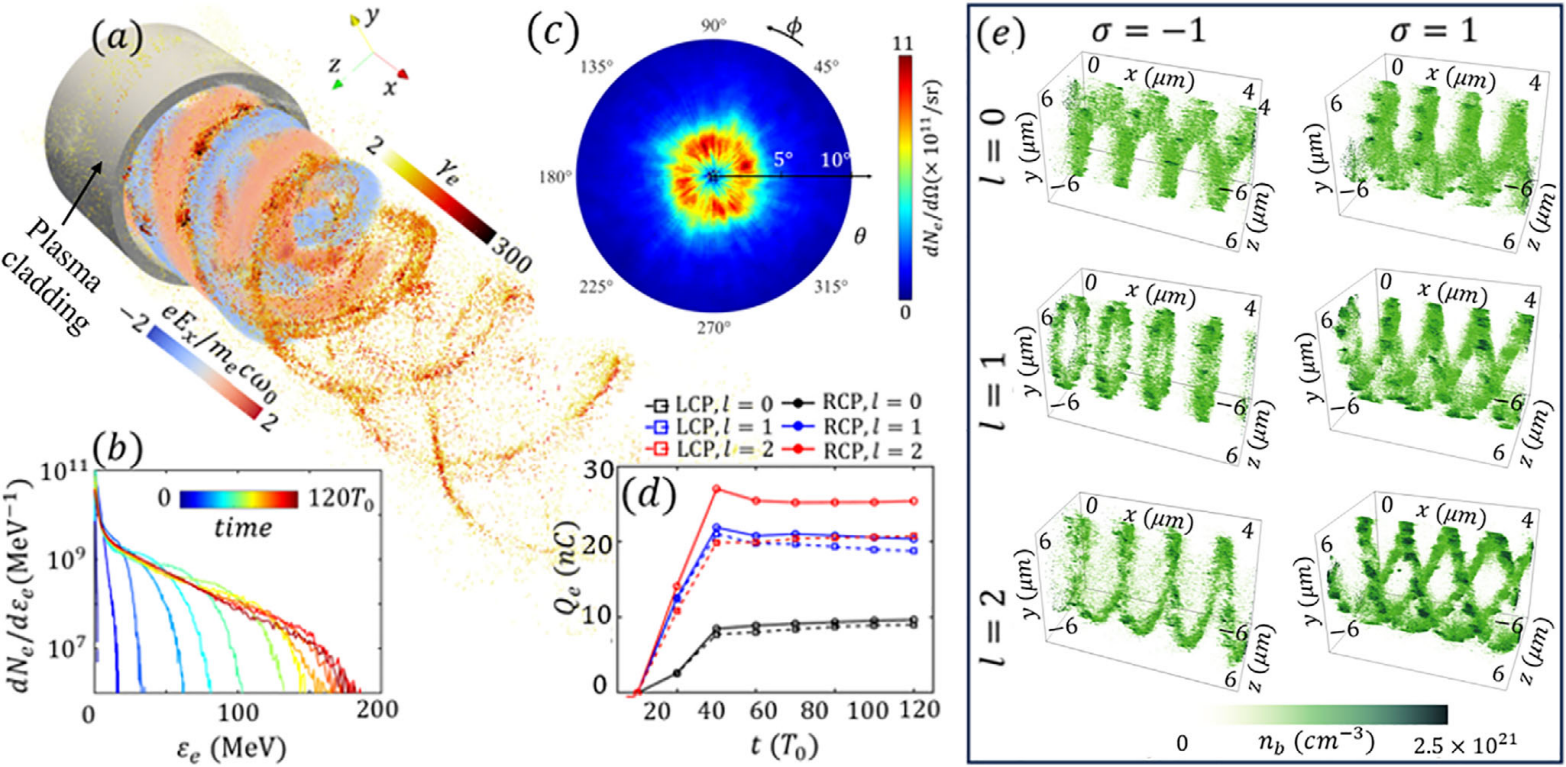
(a) The 3D structure of the accelerated electrons (color‐code with energy) and accelerating field $E_{x}$ at $t=120T_{0}$, when the electron energy reaches maximum. (b) The temporal evolution of the accelerated electron spectrum and (c) angular distribution at 50−μm ($t=170\;T_{0}$) behind the exit of the waveguide. (d) The total charge and (e) the 3D density profile of high‐energy ($\gamma_{e}>100$) electrons accelerated by drive pulses with various polarization and topological charges.

When the drive laser pulse is sufficiently strong, electrons in the plasma cladding can be pulled out, forming overdense electron bunches that can be accelerated by the longitudinal electric field [32, 38]. In Figure 2a, the injected electrons at the entrance of MPW are represented by the orange‐white dots. These electrons are rapidly boosted to relativistic energies (γ>2), enabling them to keep up with the accelerating phase ($E_{x}<0$) throughout the acceleration, until dephasing occurs. As a result, the 3D shape of the accelerating field $E_{x}$ is imprinted onto the spatial structure of the high‐energy electron beam, leading to helical micro‐bunching. This is illustrated in Figure 3a, where the electron beam structure after 120−μm of propagation in the MPW is presented, with simulation parameters identical to those in Figure 2a.

The micro‐bunching of the electron beam is expected to exhibit a helical structure (mσ=l+σ=2) as suggested by Equation (6), which agrees with the observed double‐helix beam structure. Notably, the helicity of the electron beam matches the $E_{x}$ field shape of the ${\mathrm{HE}}_{2,1}$ mode, indicating this micro‐bunching process can be controlled by the total angular momentum of the drive pulse.

The temporal evolution of the electron energy spectrum is presented in Figure 3b, which shows the electrons continuously gain energy as they propagate inside the MPW for approximately 120μm. The acceleration terminates due to dephasing between the electron and the superluminal accelerating field. This effect is apparent by comparing Figures 3a and 2a, where the electrons are injected uniformly into the accelerating bracket ($E_{x}<0$) at the entrance of MPW (Figure 2a), but they shift backwards with respect to the accelerating field as they propagate. By approximately $∼120\;T_{0}$, most electrons have fallen into the nodes of accelerating field ($E_{x}\approx 0$) (Figure 3a). Therefore, the dephasing length is approximately $L_{d}\approx 4\pi^{2}r_{0}^{2}/x_{|m|,n}^{2}\lambda_{0}$, corresponding to the relative shift between accelerating electrons and the waveguide mode reaches $\lambda_{0}/2$ [32].

Moreover, Figure 3b suggests that despite the electron energy spectrum being broadband in general, the electron charge is very high (∼10 pC/MeV at 100 MeV). This makes it possible to obtain a mono‐energetic bunch via energy filtering [52]. The angular distribution of the accelerated electron beam at 50−μm behind the exit of the waveguide is illustrated in Figure 3c. The electrons form a doughnut shape with a singularity at the MPW axis, which is a signature of helical beam. The beam divergence is around $\theta ∼2^{\circ }$, and the electron number per solid angle reaches $10^{12}\;{\mathrm{sr}}^{-1}$.

Such a high beam charge stems from the fact that electrons are extracted from the solid‐density plasma cladding of the MPW. Figure 3d indicates that the accelerated electron charge are typically on the order of tens of nC for a 100‐TW, few‐Joule class laser system. Figure 3d also shows the beam charge increases with the absolute value of topological charge |l| of the driver. This is because the electromagnetic intensity of LG drivers forms a ring, where the radius increases with |l|. Thus, the interaction between the laser and the plasma cladding is increasingly strong as |l| grows, leading to an enhanced electron injection. In addition, polarization also plays a minor role; this is probably due to high‐order radial modes, which will be discussed later.

Figure 3e displays a variety of the 3D density profile of high‐energy electrons with different helical micro‐bunching structures, as produced in our PIC simulations with different combinations of l and σ. For all the cases presented here, the azimuthal structure of the accelerating field leads to helical micro‐bunching ∼exp[i(l+σ)ϕ], consistent with our theory.

## 4 Discussion

### 4.1 Maximum Electron Acceleration Energy for High Order Waveguide Modes

In the following, we discuss the maximum electron energy excited by the high‐order waveguide modes. Previously, it was found that the electron acceleration energy is limited by the dephasing between the accelerated electrons and the superluminal phase velocity of the waveguide mode [32, 38], namely $\varepsilon_{max}\approx \left(k_{T}/k_{0}\right)E_{acc}L_{d}\propto x_{|m|,n}^{-1}a_{0}r_{0}$. A linear correlation of $\varepsilon_{max}$ with $a_{0}$ and $r_{0}$ was verified for a Gaussian drive laser beam by PIC simulations [32]. Here we focus on the effects of high‐order modes, in particular the eigenvalue $x_{|m|,n}$. However, it should be noted that the transverse motion of accelerated electrons is ignored in previous theoretical analysis of dephasing effect [32]. This is a good approximation for the fundamental mode (m=1) because the transverse size of the acceleration bracket is sufficiently large, but such an approximation may break down with increasing |m|. This is because the transverse size of each acceleration bracket shrinks with |m| as shown in Figure 2b. Therefore, the effect of electron transverse motion emerges only when |m| is sufficiently large. We show that it allows the electrons to keep up with the accelerating phases, mitigating the dephasing effect that would otherwise develop very quickly.

**FIGURE 4:**
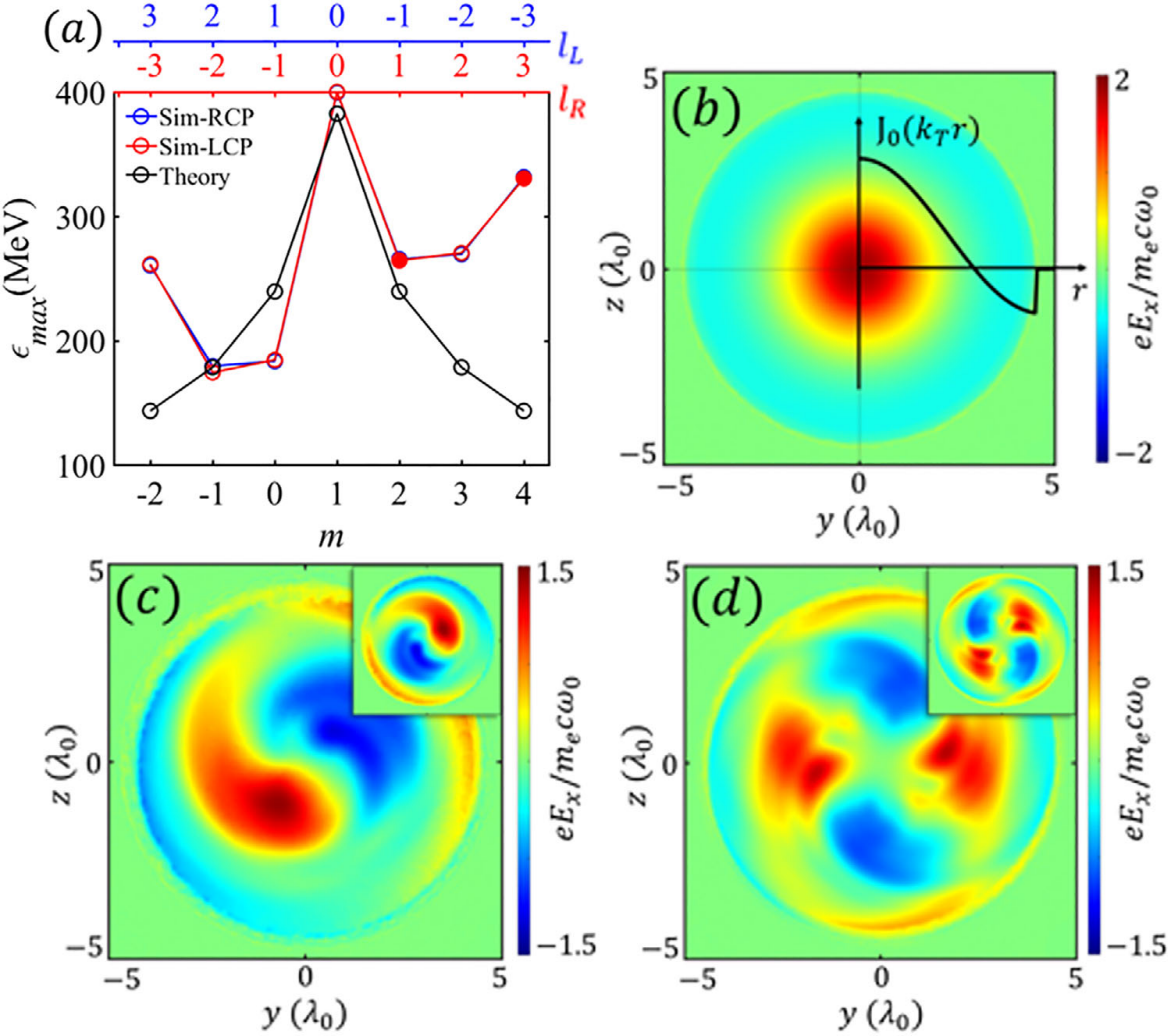
(a) The maximum energy plotted against azimuthal mode number m for various polarization and topological charges. The red and blue color represents PIC results for RCP and LCP drivers, and the black color shows the prediction of our model. (b), (c), and (d) show the acceleration field $E_{x}$ distribution with mode number m=0, −1, and −2 for RCP drivers, respectively. The black line in (b) shows the prediction of our theory, and the insets in (c, d) shows the LCP case for the same m. The solid red circles in (a) marker two cases with $l_{R}=1$ and $l_{R}=3$, where the electron trajectories are investigated in Figure 5.

To show this effect, we thus scanned l=−3 to 3 for σ=±1 in our PIC simulations; the results are presented in Figure 4a, with the same normalized laser intensity $a_{0}=15$ in each case. As can be see, the maximum electron energies are almost identical for the RCP and LCP drivers. The maximum acceleration energy is achieved at m=1, corresponding to the Gaussian laser beam (l=0). This is in agreement with our theory, since this mode has the lowest eigenvalue $x_{1,1}\approx 2.4$ as shown in Figure 1b, which leads to the longest dephasing length as expected.

It is worth noting that when m=0, the ${\mathrm{HE}}_{0,1}$ mode has a larger eigenvalue $x_{0,1}\approx 3.7$ than the ${\mathrm{HE}}_{1,1}$ mode, which leads to a faster phase velocity and a lower electron energy. Moreover, this also results in a ring shaped accelerating field ($E_{x}$) as shown in Figure 4b: comparing Equations (6) and (7), one can see that the *transverse* field of the ${\mathrm{HE}}_{1,1}$ mode and the *longitudinal* field of ${\mathrm{HE}}_{0,1}$ both have a radial dependence of $∼J_{0}\left(k_{T}r\right)$, but since the $E_{⊥}$ field is approximately zero at the boundary for the ${\mathrm{HE}}_{1,1}$ mode (see Figure 2c), the longitudinal field of the ${\mathrm{HE}}_{0,1}$ must reverse sign due to a greater transverse wavenumber $k_{T}=x_{|m|,n}/r_{0}$. Consequently, it produces a train of ring‐shaped electron bunches, as indicated by the case with l=1 and σ=−1 in Figure 3e.

The asymmetric behavior for m>0 and m<0 is counter‐intuitive, as the left‐hand side of Equation (1) is an even function of m, yet the PIC simulation results presented in Figure 4a show the electron acceleration energy is much lower on the m<0 side at the same absolute value. This is because the dominant waveguide mode generated in the m<0 cases are the second‐order radial modes (${\mathrm{HE}}_{1,2}$ and ${\mathrm{HE}}_{2,2}$ for m=−1 and m=−2, respectively), as presented in Figure 4c,d, with the signature being a sign reversal of the $E_{x}$ field as r increases (see Figure 2b for a comparison with ${\mathrm{HE}}_{1,1}$ and ${\mathrm{HE}}_{2,1}$ modes).

Finally, the most prominent feature is the substantial underestimation of the maximal acceleration energy at large |m| by the theory (the aforementioned differences in the primary radial mode orders at m>0 and m<0 sides have been considered, but transverse migration effect is not accounted for). This unexpected energy boost highlights the importance of transverse migration of the accelerating electrons.

**FIGURE 5:**
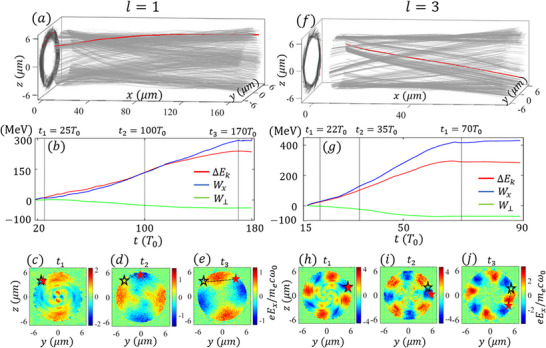
(a) The 3D trajectories of 1000 most energetic electrons accelerated by RCP LG laser beams with topological charges l=1, where the 2D projection of the trajectories are shown the (y,z) plane. (b) The work done by the longitudinal ($W_{x}$) and transverse electric field ($W_{⊥}$), as well as the energy gain ($\Delta E_{k}$) are plotted vs. time for one representative electron shown by the red color in (a). The transverse position (red star) of the representative electron superposed on the local $E_{x}$ field distribution in the same cross‐section at (c) the beginning, (d) the middle, and (e) the end of acceleration process [marked by vertical dashed lines in (b)], where the black open star marks the position where the representative electron is injected initially. (f‐j) are the same as (a–e) but for RCP LG driver with l=3.

To investigate this problem, we tracked 1000 electrons that achieved the highest energies in the PIC simulation, their trajectories are presented in Figure 5a,f for RCP LG drivers with l=1 and l=3, respectively. One can see that the electrons gain the highest energies propagate near the boundaries of the MPW throughout the acceleration, and they all shift azimuthally in the same direction as the local accelerating phase.

Since $d\varepsilon_{e}/dt=-e\left(E_{x}v_{x}+E_{⊥}\cdot v_{⊥}\right)$, one can distinguish the energy gain by tracking the work done by the transverse ($W_{⊥}=-eE_{⊥}\cdot v_{⊥}$) and longitudinal ($W_{x}=-eE_{x}v_{x}$) component of electric field throughout the acceleration, which is shown in Figure 5b,g. The representative electrons are randomly chosen in the aforementioned 1000 most energetic electrons, whose trajectories are marked by the red line in Figure 5a,f. It is evident that $W_{x}$ is dominant in both cases as illustrated in Figure 5b,g. The azimuthal shifting thus allows the electrons to keep up with the accelerating phase of the $E_{x}$ field over a long distance, which is crucial for reaching high energy.

To understand the discrepancy between the theory and simulation at large |m|, we plot the transverse positions of the tracked electrons superimposed on the local accelerating field in Figure 5c–e,h–j for different LG drivers with l=1 and l=3, respectively. The three plots show the snapshots at the beginning, middle, and the end of acceleration process as indicated by the vertical dashed lines in Figure 5b,g, which shows clear evidence of dephasing. One can see the red stars, that represent the current location of the electrons gradually fall into the decelerating phase.

However, by comparing with the initial injection positions of the electrons (the black open stars), one can see the mitigating effect on dephasing due to electron azimuthal migration. If the electrons had stayed at its initial injected position, the dephasing would occur much earlier at times corresponding to the middle plot ($t_{2}$). Apparently, this effect becomes important when the azimuthal migration angle (Δα) becomes comparable to the size of the accelerating bracket (∼π/|m|). Therefore, it can be neglected for the fundamental mode m=1, where the size of accelerating bracket is maximal, and also when m=0, because the shape of the accelerating phase is a ring, thus azimuthal migration has no effect. For high‐order waveguide modes with larger |m|,  the transverse size of each accelerating bracket is smaller. Therefore azimuthal migration effect becomes increasingly important.

**FIGURE 6:**
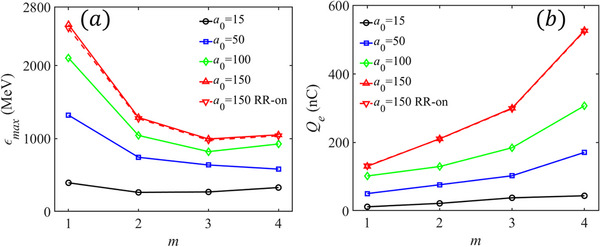
(a) The maximum electron acceleration energy and (b) the total beam charge dependence on the azimuthal waveguide mode number at different driven LG laser intensities.The red dashed line represents the simulation results that take into account the radiation reaction (RR) effect when $a_{0}=150$.

In practice, the PIC simulations suggest the azimuthal migration distances varies little, therefore the shrink of acceleration bracket with |m| is dominant factor (see Supplemental Materials). For high‐order modes that satisfy (Δα≫π/|m|), the dephasing distance is almost completely determined by azimuthal migration. This indicates that the variation of maximal acceleration energy is small when |m| is sufficiently large. This agrees with our PIC simulation results presented in Figure 6, which presents a parameter scan of the maximal acceleration energy ($\varepsilon_{max}$) and total charge ($Q_{e}$) of the beam over different laser intensities and azimuthal mode numbers m (m>0 is considered). One can see that as the value of m grows, $\varepsilon_{max}$ reduces rapidly at beginning, and then tends to saturate when m≥3. Therefore, thanks to the azimuthal migration effect, the high‐order waveguide modes that are essential for inducing arbitrary customized helical micro‐bunching of the electron beam, leads to only ∼1/2 of the cut‐off energy reduction compared to the fundamental mode.

Meanwhile, the beam charge increases rapidly up to 500 nC with m. One can see that with a 10‐PW‐level laser system, very high‐charge (100s nC), GeV helical beams can be obtained with this scheme.

Finally, as shown by red dashed lines in Figure 6a,b, the radiation reaction (RR) effect has little impact on the acceleration outcome even if $a_{0}$ is as high as 150 (laser intensity $∼3\times 10^{22}$ W/${\mathrm{cm}}^{2}$). This can be attributed to the fact that the dominant accelerating field is co‐linear to the electron motion, and the transverse force acting on the accelerated particles is negligible.

### 4.2 Waveguide Modes Generated by a Linearly‐Polarized LG Pulse

**FIGURE 7:**
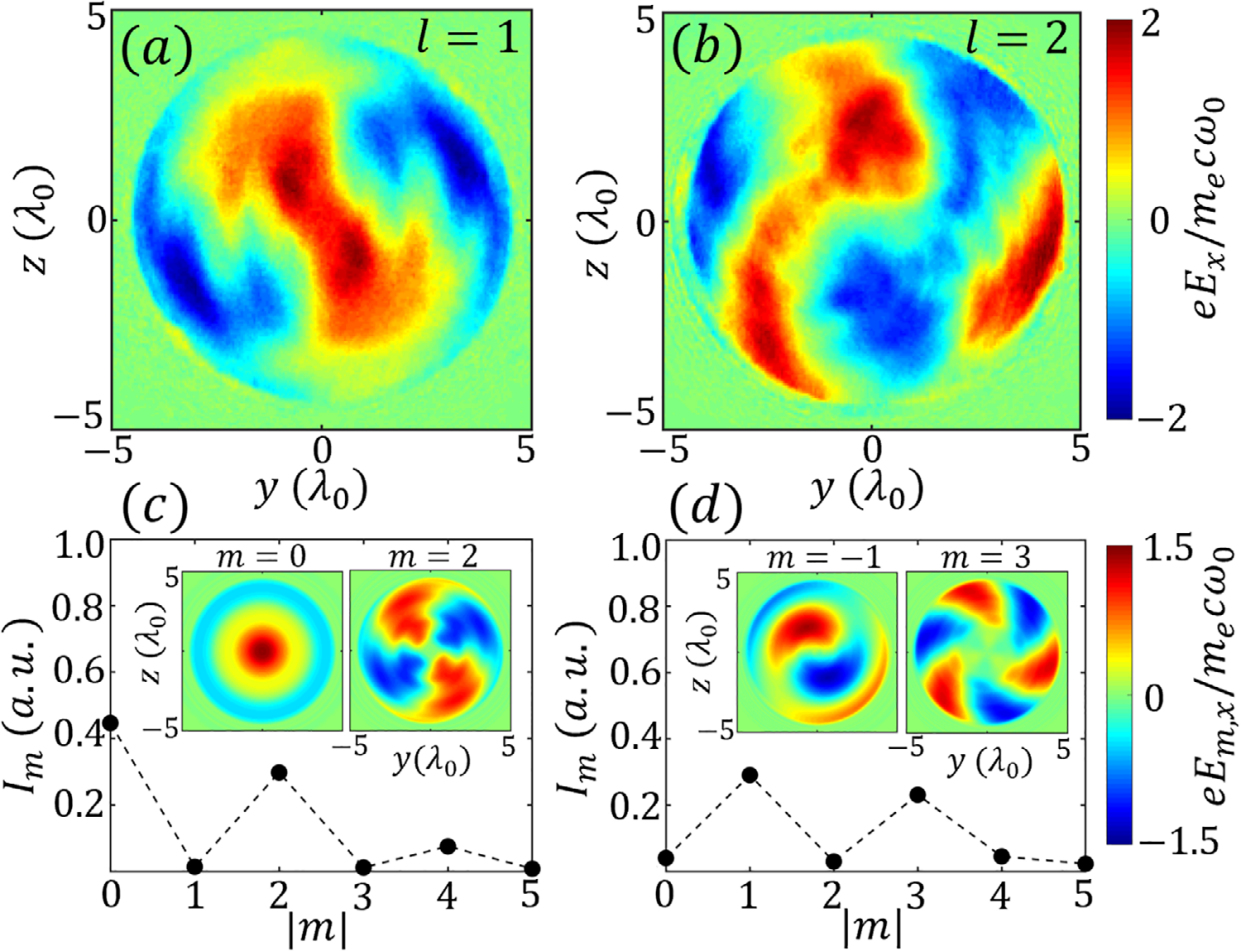
The longitudinal field distribution in the (y−z) transverse cross‐section for LP LG pulses with topological charges (a) l=1 and (b) l=2. (c) and (d) show the azimuthal spectra obtained from azimuthal Fourier transformation of (a) and (b), respectively. The insets in (c) and (d) shows the 2D field distribution of each azimuthal component in the corresponding plot.

When the incident laser pulse is linearly polarized (LP), it leads to the generation of multiple waveguide modes in the MPW, as shown in Figure 7. The longitudinal field distributions in the transverse cross‐section (y−z) are presented for LP LG pulses with topological charges l=1 and l=2 in Figure 7a,b, respectively. By applying azimuthal Fourier transformation to these fields, one can obtain their azimuthal spectra as shown in Figure 7c,d, which indicate that the electromagnetic field produced by a LG pulse with l=1 contains two components with |m|=0 and 2 (Figure 7c), and in the l=2 LP case, the electromagnetic field can be decomposed into two modes with |m|=1 and 3, respectively (Figure 7d). In both cases, the intensities of these modes are approximately the same.

The field distribution of these four modes can be obtained via inverse azimuthal Fourier transformation, which are illustrated in the insets of Figure 7c,d. The sign of m can then be inferred from the radial mode number, for the LP LG pulse with l=2, two azimuthal modes with m=−1 and m=3 are generated.

This behavior can be understood by decomposing the incident LP LG laser pulse into two CP pulses with the same topological charge l but opposite polarization handedness σ=±1. According to Equation (9), the RCP component produces a single azimuthal mode with m=l+1, and the LCP component generates a different azimuthal mode with m=−(l−1), which agrees very well with the PIC simulation results. Notably, the two modes produced from a LP LG laser have approximately the same phase velocity according to Equation (1). As a result, a LP pulse does not change its polarization angle by traveling in a plasma waveguide.

## 5 Conclusion

In conclusion, we have shown that circularly polarized LG pulses can generate high‐order azimuthal modes in a plasma waveguide. Overdense electron bunches are pulled out from the wall and accelerated by the longitudinal electric field of the waveguide modes. Therefore, the 3D helical structures of the high‐order modes are imprinted onto the high‐energy electron beams, leading to helical micro‐bunching of the beams. A high‐order waveguide mode theory is derived in this work, which shows the azimuthal mode number is m=(l+σ)σ, and the helicity of the electron bunch is controlled by the total angular momentum of the driver (mσ=l+σ). In particular, the electrons can migrate azimuthally to keep up with accelerating brackets, leading to a significant boost in the acceleration energy at large |m|. For an LP LG laser pulse, it can be decomposed into two CP components with the same l but opposite σ values, and consequently two waveguide modes are generated. These results are verified in 3D PIC simulations, which paves the way toward future tabletop helical electron beam accelerators.

## Conflicts of Interest

The authors declare no conflicts of interest.

## Supporting information


**Supporting File**: advs75489‐sup‐0001‐SuppMat.pdf.

## Data Availability

The data that support the findings of this study are available from the corresponding author upon reasonable request.
