# Supplementary material for: Helical Electron Beam Micro‐Bunching by High‐Order Modes in a Micro‐Plasma Waveguide
Source: Adv Sci (Weinh). 2026 Apr 30;13(41):e75489. doi: 10.1002/advs.75489 (PMC13335548; doi:10.1002/advs.75489)
Supplement: Supplementary file 1 — Supporting File: advs75489‐sup‐0001‐SuppMat.pdf. [file ADVS-13-e75489-s001.pdf]

Helical Electron Beam Micro-Bunching by High-Order Modes in a Micro-Plasma Waveguide——Supplemental Materials

Xingju Guo[1] and Longqing Yi[1,2]

[1]State Key Laboratory of Dark Matter Physics, Key Laboratory for Laser Plasma (Ministry of Education), Tsung-Dao Lee Institute & School of Physics and Astronomy, Shanghai Jiao Tong University, Shanghai 201210, China
[2]Collaborative Innovation Center of IFSA (CICIFSA), Shanghai Jiao Tong University, Shanghai 200240, China

1. Numerical convergence test

In order to test numerical convergence of PIC simulations, we have performed additional simulations with a grid resolution of $dx \times dy \times dz = 0.2 \times 0.04 \times 0.04\mu m^3$ per cell, and 3 macro-particles per cell for electrons (the simulation resolution adopted in our manuscript is $dx \times dy \times dz = 0.25 \times 0.05 \times 0.05\mu m^3$, and with 4 macro-electrons per cell). The convergence test results are presented in Fig. S1, which demonstrate that the time-dependent energy spectrum (average acceleration gradient), electron charge, and helical microbunching are consistent against different simulation resolutions.

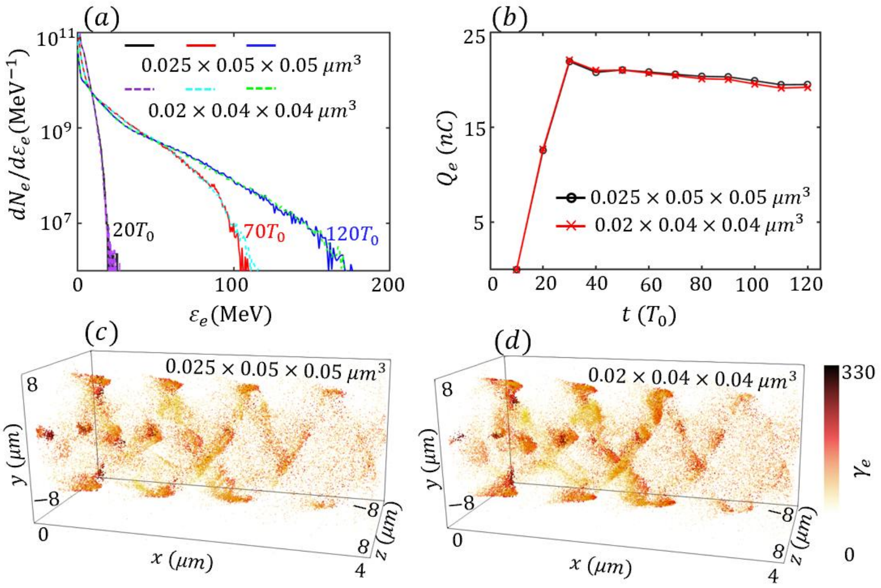

Fig S1 Numerical convergence test: (a) time-dependent electron energy spectra, (b) total electron beam charge as well as the (c-d) 3D micro-bunching structures from PIC simulations with two different resolutions: $dx \times dy \times dz = 0.25 \times 0.05 \times 0.05\mu m^3$

and $dx \times dy \times dz = 0.2 \times 0.04 \times 0.04\mu m^3$. The laser and plasma parameters are the same as in Fig. 2 and Fig. 3 in the manuscript, and the numerical resolutions are marked in the corresponding figures.

2. Discussions on the high-order radial modes

In general, high-order radial modes are generated due to mismatch at the entrance of MPW. However, we believe that the excitation of high-order radial modes does not affect our key results regarding the acceleration process, for the following reasons.

First, the laser energy coupled into high-order radial mode is relatively small compare to the fundamental mode. As shown by Fig. S2 below, it can be estimated from the longitudinal electric field pattern that the intensity ratio between the fundamental radial mode (EH_{21}) and the second radial mode (EH_{22}) is approximately 4:1.

Second, and more importantly, since all the modes excited in the MPW have the same azimuthal mode number, the lowest radial mode has the smallest eigenvalue, corresponding to lower phase velocity (and greater group velocity). This can be inferred from Fig. S2(c) that after propagating in the MPW for 120 T_0, the EH_{22} mode falls behind, and the electric field is almost pure EH_{21} mode. Therefore, the fundamental mode has the longest dephasing distance and consequently leads to maximum acceleration. Thus, it is reasonable to consider only the lowest radial mode as we are interested in the cut-off energy of MPW electron acceleration. In other words, the contribution of high-order radial modes to the electron energy is mostly canceled out in the acceleration length scale of interest (i.e. the dephasing length of the lowest radial mode).

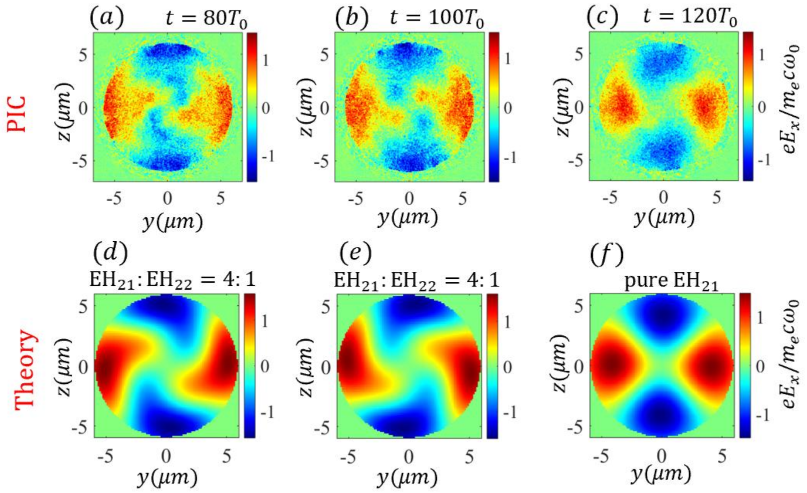

Fig S2 The longitudinal electric field pattern at the peak of the drive laser pulse observed in PIC simulations at (a) $t = 80T_0$ (b) $100T_0$, and (c) $120T_0$; as well as the field pattern reconstructed by the waveguide mode theory considering the fundamental radial mode (EH_{21}) and the second radial mode (EH_{21}).

3. Statistics on the electron transverse migration distances

In order to study how the transverse migration affect electron acceleration by the high-order azimuthal modes, we plot the number of energetic electrons as a function of their maximum transverse migration distances in Fig. S3. One can see that the average migration distance is very similar for these two cases, $\overline{D}_m = 5.7\mu m$, $4.8\mu m$, and $4.7\mu m$ for $l = 1$, 2, and 3, respectively. The average migration distance for the $l = 1$ case is slightly higher, but the root mean square is also larger.

Therefore, we conclude the migration angles $\Delta\alpha \sim D_m/r_0$ are similar, and the transverse migration effect is important for high order modes is because of the shrink of accelerating bracket with $|m|$. A more detailed study on the quantitative characteristic of the relation between electron migration (angle, velocity, etc) and mode number m is outside the scope of current work, and should be left for further study.

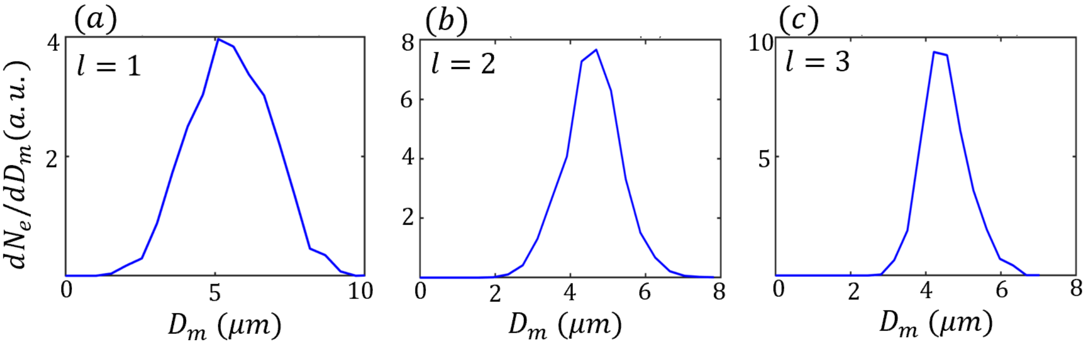

Fig. S3 The number of energetic electrons as a function of their maximum transverse migration distance for RCP drive laser pulses with (a) $l = 1$ and (b) $l = 2$ and (c) $l = 3$. Here we consider 3000 macro-electrons that achieved maximum acceleration energy in each simulation.
